# Investigating the flow of information during speaking: the impact of morpho-phonological, associative, and categorical picture distractors on picture naming

**DOI:** 10.3389/fpsyg.2015.01540

**Published:** 2015-10-12

**Authors:** Jens Bölte, Andrea Böhl, Christian Dobel, Pienie Zwitserlood

**Affiliations:** ^1^Institut für Psychologie, Westfälische Wilhelms-Universität MünsterMünster, Germany; ^2^Institut für Lernsysteme GmbH, HamburgGermany; ^3^HNO Klinik, Universitätsklinikum JenaJena, Germany

**Keywords:** picture–picture paradigm, morphology, spoken word production, cascading activation, discrete activation, semantic relatedness, assoicative relatedness, categorical relatedness

## Abstract

In three experiments, participants named target pictures by means of German compound words (e.g., *Gartenstuhl*–garden chair), each accompanied by two different distractor pictures (e.g., lawn mower and swimming pool). Targets and distractor pictures were semantically related either associatively (garden chair and lawn mower) or by a shared semantic category (garden chair and wardrobe). Within each type of semantic relation, target and distractor pictures either shared morpho-phonological (word-form) information (*Gartenstuhl* with *Gartenzwerg*, garden gnome, and *Gartenschlauch*, garden hose) or not. A condition with two completely unrelated pictures served as baseline. Target naming was facilitated when distractor and target pictures were morpho-phonologically related. This is clear evidence for the activation of word-form information of distractor pictures. Effects were larger for associatively than for categorically related distractors and targets, which constitute evidence for lexical competition. Mere categorical relatedness, in the absence of morpho-phonological overlap, resulted in null effects (Experiments 1 and 2), and only speeded target naming when effects reflect only conceptual, but not lexical processing (Experiment 3). Given that distractor pictures activate their word forms, the data cannot be easily reconciled with discrete serial models. The results fit well with models that allow information to cascade forward from conceptual to word-form levels.

## Spoken Word Production

The production of a simple greeting such as “Hi” is the result of series of cognitive processes that precede articulation. Processes such as conceptualization, message generation, lexical selection, morpho-phonological processing, phonetic encoding, and monitoring all take place prior to articulation (Dell, 1986; Butterworth, 1989; Levelt, 1989; Levelt et al., 1999). How information flows between how many different processing levels is a much-debated topic, distinguishing between serial-discrete (“two-step#x201D;) models, fully cascading models and fully interactive models (see Levelt, 1989). Interactive models allow for bidirectional information flow (from conceptual to phonological information, and vice versa). The major difference between discrete and fully cascading models concerns the information that is activated at certain processing stages, which are detailed below.

In the current study, we tested predictions derived from discrete and fully cascading models. We assessed the flow of information during speaking by investigating how distractor pictures that are not targets for speech production influence the speed with which a target picture is named. We varied the relationship between the distractor and target pictures to assess how “deeply” distractor pictures are processed. Target and distractor pictures could be semantically related (target “sunbed”, distractors “beach ball”, and “flippers”), and in addition, their names could share a morpheme (target “sheepdog”, distractors “sheep pen”, and “sheep wool”). An impact of these types of relatedness on picture naming is informative about the flow of information in speech production. To elucidate different predictions by the models that are put to test here, we briefly sketch these models.

Models of speech production agree that speaking makes demands on the following types of information. The first, conceptual/semantic information of the to-be-expressed concepts is often considered not to be lexical but part of semantic memory. Lexical information consists of grammatical aspects (e.g., word class, gender) and information about the form of words, including their morphological make up (cf. “collie” and “sheepdog”) and phonological specification (e.g., /d/ /o/ /g/). But models disagree with respect to the processing flow from conceptual to phonological information. In the serial models (Garrett, 1980; Levelt et al., 1999), speaking proceeds serially, in ordered steps, from conceptual processing to articulation. Critically, there are two distinct steps; the first step allows cascading of information, such that many representations can be active at adjacent levels of processing. The second step is only initiated when a selection process has delivered a single, complete output (cf. Levelt, 1989; Roelofs, 1997; Levelt et al., 1999; Bloem and La Heij, 2003). In discrete, two-step models, concepts activate multiple lexical entries at an initial level, labeled “lemma level”. Lemmas code the grammatical features (word class, gender, and so on), but not the morpho-phonological make up of lexical entries. Many related concepts (*dog, cat, collie*) can be active during speech production, and the activation cascades to their corresponding lemmas. Which lexical entry will be uttered is decided at the lemma level, by means of a competitive selection process (Roelofs, 1992). Selection is more difficult/takes more time when co-activated lemmas come from the same semantic category as the target (e.g., lemon–orange), because they compete more for selection than unrelated entries, or than related entries that have less semantic overlap (e.g., lemon–sour). Selection of one lemma as the target for production implies that only one lexical entry will activate its morpho-phonological word-form, and this is where cascading comes to a halt^1^.

In contrast, processing stages in fully cascading models, although temporally ordered, deliver multiple, even partial, outputs to consecutive stages, allowing for the simultaneous activation of many word forms (Dell, 1986). Some of these models do not adopt a separate lemma level (Stemberger, 1985; Humphreys et al., 1988; Caramazza, 1997; Peterson and Savoy, 1998). The selection as to which word will be uttered is non-competitive; to cite Mahon et al. (2007, p. 203) “the level of activation of a non-target does not affect the selection of the target”. Thus, there are two crucial differences between these models; (1) discrete, two-step models predict interference, reflecting competition during selection due to the presence of same-category stimuli, but fully cascading models do not and (2) cascading models allow and predict that word-form (morphological and phonological) information is simultaneously available for more than one lexical entry, but discrete two-step models do not. Jescheniak and Schriefers (1998), Peterson and Savoy (1998), Rapp and Goldrick (2000), as well as Goldrick (2006) offer overviews of the discrete/cascading controversy.

## Cascaded or Discrete Processing, Paradigms, and Evidence

In the following, we summarize the evidence in favor of fully cascaded, and against discrete, processing in speech production, and introduce the paradigms used together with their basic findings. Next, we present the manipulations and predictions for the three experiments of our study.

So far, evidence for cascaded processing comes from (1) speech errors, (2) picture naming experiments with word distractors, and (3) picture-naming experiments with picture distractors – the paradigm that we also used here. Speech-error data from patients and simulations of speech-error data argue against discrete models (Rapp and Goldrick, 2000). The relevant error type concerns mixed errors. A mixed error is a word that is semantically and phonologically related to the intended word (e.g., saying *cat* instead of *calf*). Taking error distributions into account, such errors are more likely to occur than pure semantic errors (e.g., saying *dog* instead of *cat*; Dell and Reich, 1981; Martin et al., 1996). Rapp and Goldrick (2000) argue that mixed errors can only occur in fully cascading models and/or interactive models, but not in discrete serial models. Roelofs (2004), however, argues that mixed errors result from erroneously selecting two lemmas instead of one. In his view, erroneous selection of multiple lemmas is not restricted to mixed errors but is also the basis for blend errors (e.g., *close* + *near* →*clear*, cf. Roelofs, 1992) and for activating multiple word forms of near synonyms.

The next source of evidence comes from picture–word interference (PWI) studies. In paradigms with word distractors, a picture that has to be named is accompanied by (written or spoken) words that can be ignored. Such PWI studies consistently show that picture naming is faster when distractor words are related in form (picture of a calf, distractor “cart”) than when not (picture of a calf, distractor word “bowl”; Meyer and Schriefers, 1991; Levelt et al., 1999, for an overview). This also holds for cases of large form overlap, when target and distractor word share a morpheme (picture of a sheepdog, distractor “sheep wool”), even when there is no obvious semantic relation between the concepts specified by picture and distractor word (e.g., picture of a hummingbird, distractor “jailbird”; see Lüttmann et al., 2011a). Semantically related distractors that do not share the target’s semantic category (picture of a cow, distractor “milk”) tend to speed target naming. This is often interpreted as stemming from the non-lexical, conceptual level (see La Heij et al., 1990; Alario et al., 2000). However, picture naming is slowed when the distractor comes from the same semantic category as the target (picture of a calf, distractor “sheep”). This is interpreted either as evidence for competitive lexical selection (Schriefers et al., 1990; Roelofs, 1992; Levelt et al., 1999), or as originating from post-lexical problems, occurring when a semantically related distractor word occupies a prominent place in the serial output buffer, thus hindering the timely output of the picture name (Mahon et al., 2007).

With respect to the issue of full or partial cascading, experiments with word distractors that are related in both meaning and form to the target picture (e.g., target picture *calf, distractor word “cat”*) revealed interactive effects: form relatedness counteracts the negative consequences of a shared semantic category between target and distractor (Starreveld and La Heij, 1995, 1996; Damian and Martin, 1999). Moreover, near synonyms or cognates (for bilinguals) activate multiple word forms (Jescheniak and Schriefers, 1998; Peterson and Savoy, 1998; Costa et al., 2000), also supporting the notion of full cascading.

Finally, some studies using multiple pictures instead of pictures and words also argue for a continuous cascade of information. In picture–picture paradigms, a target picture for naming is accompanied by one or more distractor pictures that should not be named (Glaser and Glaser, 1989; Morsella and Miozzo, 2002; Damian and Bowers, 2003; Navarrete and Costa, 2005; Meyer and Damian, 2007; Oppermann et al., 2008, 2014; Roelofs, 2008a). Morsella and Miozzo (2002) asked their participants to name one of two differently colored, superimposed line drawings, and to ignore the other. Faster picture-naming latencies were obtained for phonologically related (*bed-bell*) than for unrelated pictures (*hat-bell*; see also Damian and Bowers, 2003; Navarrete and Costa, 2005; Meyer and Damian, 2007; Roelofs, 2008a), suggesting that the distractor picture activates its phonological representation, which then (because of phonological overlap) speeds up target naming. Jescheniak et al. (2009), who failed to replicate this data pattern, suggest that differences in amount of phonological overlap, the inclusion of the distractor pictures in the response set, and/or subtle differences in name agreement might be responsible for the divergent results. Importantly, and despite the absence of semantic effects in Morsella and Miozzo (2002), the presence of phonological effects argues for the full cascading of activation.

The absence of semantic effects (e.g., *table–bed*) in Morsella and Miozzo (2002) is rather startling, given that language production proceeds from semantic to phonological representations. In general, studies using picture–picture paradigms showed diverging results for categorically related distractor pictures: facilitation (Bloem and La Heij, 2003; Roelofs, 2008a), interference (Glaser and Glaser, 1989), or no effects (Humphreys et al., 1995; Morsella and Miozzo, 2002; Damian and Bowers, 2003; Navarrete and Costa, 2005). It is not yet fully understood what causes the different result patterns. With picture distractors, it does not seem mandatory that all available conceptual information is automatically encoded lexically, and the task, target set, attention to the distractor picture, and material manipulations might play an important role.

One important factor concerns the availability of distractor pictures as (potential) targets – sometimes manipulated by including all pictures in the target set. This fits with data from Aristei et al. (2012), who presented two pictures simultaneously that both had to be named to produce a novel compound (e.g., *lion dog*). Participants were slower in producing such novel noun–noun compounds when the two pictures were categorically related (*lion dog*) than when not (*chair dog*). Aristei et al. (2012) argue that this provides evidence for lexical competition.

Similar conclusions can be drawn from studies by Oppermann et al. (2008, 2014), who presented a target and a distractor picture simultaneously, while spoken words that were semantically related, phonologically related or unrelated to the distractor picture served as additional distractors. When target and distractor objects were similar in shape, semantically related distractor words slowed down target picture naming relative to unrelated distractor words. This suggests that the concepts of the target and distractor pictures enter the lexicalization process provided that distractor pictures capture sufficient activation, because they are similar in shape to the target and are “boosted” by related distractor words.

Thus, whether semantic effects can be registered in picture–picture paradigms seems to depend on the amount of attention to the distractor picture (Jescheniak et al., 2014), on how to signal the target picture and/or on the particular task implemented (Glaser and Glaser, 1989; Bloem and La Heij, 2003; Damian and Bowers, 2003).

Note that evidence for cascading semantic information *per se* does not distinguish between fully cascading and discrete, two-step models, but the direction of semantic effects (facilitation, interference) does. Interference, due to same-category membership of distractors and targets, is predicted by two-step models but not by fully cascading models. It plays an important role in the discussion about lexical-competition (discrete models), and fully cascading models provide an explanation of such interference effects in terms of a post-lexical response-buffer. We will discuss this further below.

### The Picture–Picture Paradigm, Conditions, and Predictions

To further test the predictions of discrete and fully cascading models, we opted for the picture–picture paradigm, because its suitability to test for activation of lexical form (morphology, phonology) of non-target pictures. We presented three different pictures, one of which was the target for naming. Which picture had to be named was either signaled by a cue that appeared with varying delays (Experiments 1 and 2), or was unequivocally signaled by presenting the target picture with some delay after the non-target (distractor) pictures (Experiment 3). We used multiple distractors (1) because effects can be larger with two than with one distractor (Melinger and Abdel Rahman, 2004) and (2) to create more uncertainty as to which picture has to be named eventually.

A first manipulation concerned the nature of semantic overlap between distractor and target pictures, which was either associative or categorical. Note that both models allow for the activation of multiple concepts (of all three pictures). To our knowledge, associatively related distractors (e.g., *sailor* and *ship*) or distractors representing semantic features of the target object (e.g., *porthole* and *ship*) have not been investigated so far within the picture–picture paradigm. It is well established that associatively and categorically related distractors have different effects in the PWI paradigm (Bölte et al., 2003, 2005; Costa et al., 2005; Mahon et al., 2007). Why words that are semantically associated or that represent semantic features of the target picture facilitate, whereas words that specify a same category member inhibit picture naming, is still a matter of intense debate (see Costa et al., 2005; Mahon et al., 2007; Abdel Rahman and Melinger, 2009; Janssen, 2013; Roelofs et al., 2013; Mahon and Navarrete, 2014). Whereas both associative and categorical similarity should induce priming at the level of conceptual representations, they seem to differ at lexical or post-lexical levels. According to discrete models, the activated lemmas of same-category concepts cause havoc during the selection of the lexical entry that is the target for speaking (Roelofs, 1992; Levelt et al., 1999), because they are confusable with the target and seem such valid responses (saying “dog” to a picture of a cat is more likely than saying “purr”). If we obtain categorical competition effects in a picture–picture paradigm, this is clear evidence for the existence of a competitive lexical selection process, and argues against prominent cascading models (Caramazza, 1997). Note that categorical interference from pictures also speaks against the response-exclusion hypothesis (Finkbeiner and Caramazza, 2006; Mahon et al., 2007). According to this hypothesis, the interference by categorically related distractor words observed in PWI is due to the fact that these distractors, because they are words, enter the articulatory response buffer that channels verbal responses for output. Words that are semantically related to the correct response (the picture name) are harder to remove from this buffer than unrelated words, hence, the interference. Most importantly, this holds for verbal stimuli only, not for pictures (see Jescheniak et al., 2014).

As stated above, discrete and fully cascading models also make different predictions concerning the impact of morpho-phonologically related distractor pictures on the speed of target-picture naming. We used German compound words, as distractors (*garden hose, garden gnome*) and targets (*garden chair*), because such stimuli have the advantage of sharing both semantic and form information. We crossed the type of semantic relation (associative vs. categorical) with form overlap, in terms of shared morphemes (initial or final morphemes of compound names). To our knowledge, combining semantic and form overlap has not been done before with the picture–picture paradigm (not even with partial overlap, as in “cart” and “calf”). The critical evidence for full cascading is when distractor pictures also activate their word-form information. This should not be the case according to discrete, two-step models.

As stated earlier, form-relatedness has been reliably demonstrated with the PWI paradigm, when a target picture (e.g., of a *football*) is accompanied by a distractor word that shares phonemes or morphemes with the target (e.g., “foodstuff” or “footstool”; cf. Meyer and Schriefers, 1991; Zwitserlood et al., 2002; Lüttmann et al., 2011a). In picture–word paradigms, distractor words automatically activate lexical information. Their processing proceeds from phonemes or graphemes via word-form and syntactic information to concepts. Word distractors can thus influence picture naming at all (lexical) levels. This is different for picture distractors that can only influence the lexical processing of the target if the distractors themselves activate their lexical information. Thus, if naming a “football” is easier when the distractor pictures show a “footprint” and a “footstool”, this provides clear evidence for the activation of morpho-phonological information belonging to the distractor pictures, and for full cascading of information during speech production. In contrast, the lack of activation of the distractor pictures’ word forms supports discrete, only partially cascaded models.

We thus included the following target-distractor conditions in our study. The relation between a target picture (e.g., a *garden chair*)^2^ and its two different distractor pictures was either (1) associative with morpho-phonological^3^ overlap (+A+M) in the first constituent (e.g., *garden hose, garden gnome*), (2) same-category combined with morpho-phonological overlap +C+M) in the second constituent (e.g., *rocking chair, office chair*), (3) merely associative (+A–M; e.g., a *swimming pool, lawn mower*) or (4) merely categorically related (+C–M; e.g., *office desk, shoe rack*), thus without morpho-phonological overlap, or (5) completely unrelated (e.g., *billiard ball, sock suspender*).

Our rationale to use both types of semantic relation is as follows: if effects in the picture–picture paradigm solely originate at a conceptual level, effects should be similar for categorically and associatively related distractors. If interference – or reduced facilitation, relative to associatively related pictures – is observed for categorical distractors, this is evidence for their lexical coding. Such effects provide clear evidence for competitive lexical selection (cf. Levelt et al., 1999), and against fully cascading models as well as against the response-exclusion hypothesis that only applies to words, not to pictures (Mahon et al., 2007). Note that reliable interference due to categorically related context pictures has rarely been observed in picture–picture studies reported so far, which either suggests that distractor pictures are not lexically coded automatically (cf. Damian and Bowers, 2003; Jescheniak et al., 2014), or that conceptual facilitation and lexical competition cancel each other out.

We also implemented the distinction between same category and association with pictures whose names are morpho-phonologically related to the target picture’s name. Morphological relatedness is not specified at the conceptual level (Caramazza et al., 1988; Levelt et al., 1999; Janssen et al., 2008). If all effects are conceptual, without any lexical involvement, these should behave in the same way as associatively or categorically related pictures whose name is morpho-phonologically unrelated to the target. If distractor pictures are lexically processed, but at the lemma level only (in discrete models), the same predictions hold as formulated above for morphologically unrelated distractors. But if distractor pictures are processed all the way down to their word-form level, where morphology is specified, we expect facilitation due to morpho-phonological relatedness. In PWI studies, where form effects are obvious because the distractors are words, facilitation was observed with distractors and targets overlapping at word onset and offset, both with monomorphemic words (e.g., *power* and *towel* with the picture of a *tower*) and with morphologically related (e.g., *tea rose* and *rosebush* with the picture of a *rose*) distractors (Meyer and Schriefers, 1991; Zwitserlood, 1994; Zwitserlood et al., 2002; Belke, 2005; Lüttmann et al., 2011b).

When distractor pictures are encoded at the level of word form, we expect additional facilitation due to shared morphemes, relative to an unrelated baseline, in both morpho-phonological conditions (+A+M and +C+M). The size of effects might differ because of lexical competition in the +C+M condition. The purely associatively related distractor condition (+A–M) that does not induce much lexical competition should also reveal facilitation, but the categorically related distractors (+C–M) should show no effect or even interference. This is because they are conceptually related to the target (resulting in facilitation) but also lead to interference due to lexical competition with the target. Keep in mind that the presence of interference, or reduced facilitation, in the +C conditions speaks for competitive lexical selection (Levelt et al., 1999), but is incompatible with full cascading models (Caramazza, 1997) and with response-exclusion (Mahon et al., 2007).

Finally, we manipulated the signaling of the target picture, either by a cue (an arrow, Experiments 1 and 2) or by a time delay (Experiment 3). We varied the onset of the target cue (Experiments 1 and 2) relative to the stimuli display (SOA). This had two functions. First, given that it is unclear whether multiple pictures automatically activate their lexical information, a longer uncertainly as to which picture has to be named (implemented by a larger SOA) might invite a lexical activation of all pictures. A large SOA might invite the lexical coding of more than one picture, but a small SOA should not.

The next issue concerns the time course of lexical activation. In the PWI paradigm, the impact of semantic and phonological distractors on picture naming depends on the temporal relation between word distractor and target. Categorical and associative effects are largest if the distractor precedes the target, while phonological effects arise when the distractor follows the target or is presented simultaneously with the target (Glaser and Düngelhoff, 1984; Schriefers et al., 1990; Meyer and Schriefers, 1991; Alario et al., 2000; Jescheniak et al., 2005). Similarly, providing more or less time before it becomes clear which picture is to be named might lead to the involvement of different processing levels. An SOA of 200 ms between the onset of the pictures and the cue may well be too short for the activation of word-form information, but an SOA of 600 ms should suffice. So, the SOA manipulation was used to invite or discourage the (strategic) lexical coding of all (or some) pictures before the target was signaled. In Experiment 3, it was clear to the participants that the two objects that appeared first were never to be named, because the target was signaled by means of an onset delay. In this case, lexical activation of distractor pictures might be completely absent.

We also monitored eye-movements, in addition to voice-key latencies. The reason was to investigate whether targets had to be fixated for correct naming, and whether distractors had to be attended overtly to affect target naming. Previous research using eye-movements required their participants to name all displayed objects (cf. Meyer et al., 1998). In such tasks, participants look at the object until its phonological form is planned. On the other hand, Dobel et al. (2007) showed that fixations of scene elements are not necessary to identify (and name) agents, actions and patients of action scenes. Unlike in the study by Meyer et al. (1998), participants were not asked to give speeded responses, and sometimes were even prevented from making eye-movements into the scene, because of very short scene presentation durations. So, speakers can name visual stimuli without overt attention, but they may well look at objects to facilitate object recognition and name retrieval (Meyer et al., 2012). It is still unknown whether distractor pictures have to be fixated at all to affect target naming.

## Experiment 1: Cue Onset 600 ms

### Method

#### Participants

Forty participants from the Westfälische Wilhelms-University of Münster took part in the experiment. They were either paid 4 € or received course-credit for their participation. All had normal or corrected-to-normal vision and were native speakers of German.

#### Material

We used pictures that are named with noun–noun compounds to implement the morpho-phonological similarity, concurrent with semantic similarity, between target and distractor pictures. Material selection was a multi-phased procedure. First, we selected noun–noun compounds from the Celex lexical database, discarding all compounds that were not depictable (Baayen et al., 1993). Next, distractors were constructed for each target (*Gartenstuhl*, lawn chair) such that there were three to five distractors per Distractor Type: (1) +A+M, associatively and morpho-phonologically related (e.g., *Gartenzwerg*, garden gnome; *Gartenschlauch*, garden hose), (2) +C+M, categorically and morpho-phonologically related (e.g., *Schaukelstuhl*, rocking chair; *Bürostuhl*, office chair), (3) +A–M, associatively but not morpho-phonologically related (*Rasenmäher*, lawn mower; *Schwimmbecken*, swimming pool) and (4) +C–M, categorically but not morpho-phonologically related (e.g., *Schreibtisch*, desk; *Schuhregal*, shoe rack), and finally, control distractors that were neither categorically, associatively nor morpho-phonologically related to the target (e.g., *Zahnbürste*, tooth brush; *Billardkugel*; billiard ball). This resulted in a set of 377 compounds (22 targets, 355 potential distractors). Colored pictures for these compounds were taken from the Hemera Photo Objects (n.d.) database, or from the internet.

The material was tested in two pretests: (1) an oﬄine name-agreement test in combination with a semantic rating task and (2) an online name-agreement test. Twenty participants took part in the oﬄine tests, another 15 served in the online test. All participants came from the same population as mentioned above and received a similar compensation. In the oﬄine name agreement test, each distractor picture was presented alongside its target picture, resulting in 355 trials. Participants were asked to write the word that described best the depicted objects and to rate their semantic relatedness, using a 5-point scale (1 = unrelated, 5 = related). The online name agreement test served to assess the preferred naming of the picture under conditions similar to the actual experiment (see **Table 1** for relevant means and SDs). Trials in this test were structured as follows: a fixation cross appeared on a computer screen for 250 ms, followed by the picture that remained on the screen for 600 ms Time-out was set to 1500. Participants were asked to name the picture as quickly as possible.

**Table 1:** Semantic relatedness rating and name agreement data from off- and online tasks, as a function of distractor condition (SD in parentheses).

		Percentage name agreement	
	Semantic relatedness rating	Oﬄine task	Online task
+A+M	3.9 (0.9)	92.0 (11.6)	85.5 (13.6)
+C+M	3.3 (1.0)	87.2 (14.5)	82.7 (15.1)
+A–M	3.4 (0.7)	90.7 (10.6)	78.9 (14.1)
+C–M	3.1 (0.5)	88.4 (13.2)	80.4 (14.4)
Unrelated	1.21 (0.2)	97.5 (5.2)	88.4 (10.9)

We selected all pictures that were predominantly named with a morphologically complex word in the oﬄine (targets mean: 79%, SD: 6, range: 70–85%; distractors mean: 91%, SD: 12, range: 55–100%) as well as in the online naming test (targets: mean: 81%, SD: 14, range: 60–100%; distractors: mean: 84%, SD:14, range: 53–100%). This resulted in 15 target pictures, each with two different distractor pictures in each of the five distractor conditions. Mean ratings of all pretests for the selected items are provided in **Table 1**. The semantic relatedness judgments were evaluated with the help of a one-way univariate repeated measures ANOVA over items, using semantic relatedness judgments as dependent variable and Condition (+A+M, +C+M, +A–M, +C–M) as factor. The main effect Condition was not significant [*F*_(3,42)_ = 2.172, *MSE* = 0.758, *p* = 0.105, $\eta_{g}^{2}$ = 0.117].

Targets and distractors were distributed over five lists, with list order counter-balanced across participants. Participants were presented with all lists. An additional 24 filler trials, each with pictures of three morphologically complex but unrelated words, were included in each list, to increase the number of unrelated trials (e.g., *Schlittschuh*, ice skate; *Bohrmaschine*, drilling machine; *Sonnenblume*, sunflower). Each block consisted of 39 trials plus six warm-up trials.

#### Apparatus

Pictures (ranging from 22 × 245 pixel for “toothbrush” to 241 × 207 pixel for “oil lamp”) were presented on a 21-inch Samsung SyncMaster 1100p plus CRT monitor (1024 × 768 pixel, frame rate: 85 Hz), controlled by a Dell-Dimension 4200 IBM-compatible PC. Participants were seated approximately 60 cm in front of the monitor. Eye-movements were recorded with an Eyelink II (2004) eye-tracker, with a sampling rate of 500 Hz and an eye position resolution of less than 0.5°. The eye-tracker was controlled by a Dell-OptiPlex 280. Onset naming latencies were recorded with a voice key.

#### Procedure

Participants were tested individually in a quiet room. They received a written instruction. They were informed that three pictures would appear on the screen and that shortly after picture onset an arrow would signal the picture that they had to name. Participants were asked to name the target picture as quickly and accurately as possible such that the experimenter could correctly identify the target among the other objects on the display (see Bölte et al., 2009). Before the experiment proper, the following steps were taken. First, to minimize target name variation, participants received a booklet with target pictures and names. Second, after having read the booklet, each target picture was presented again for naming on the computer screen. Third, the eyetracker was calibrated and validated using a nine-point calibration type (HV9). Upon successful validation, the experiment started. A drift-correction was applied before each trial using the fixation point.

Trial structure was as follows: a fixation point, centered in the middle of the screen, indicated the beginning of a new trial. After successful fixation, the trial began and three pictures appeared in one of four possible configurations. Either there was one picture left of, one right of (160 pixel away from screen center) and one above (or below) the fixation point (150 pixel away from screen center) or one above, one below and one left (or right) of the fixation point (6.9° apart). An arrow appeared 600 ms after picture onset, signaling the target object. Target position on a list was (nearly) equally distributed (10 top, 10 left, 10 right, 9 bottom). Pictures disappeared with the participants’ voice onset or after 5000 ms. Stimuli were presented as colored photographs on a white background. The experimenter wrote down the participants’ answers.

### Results

**FIGURE 1:**
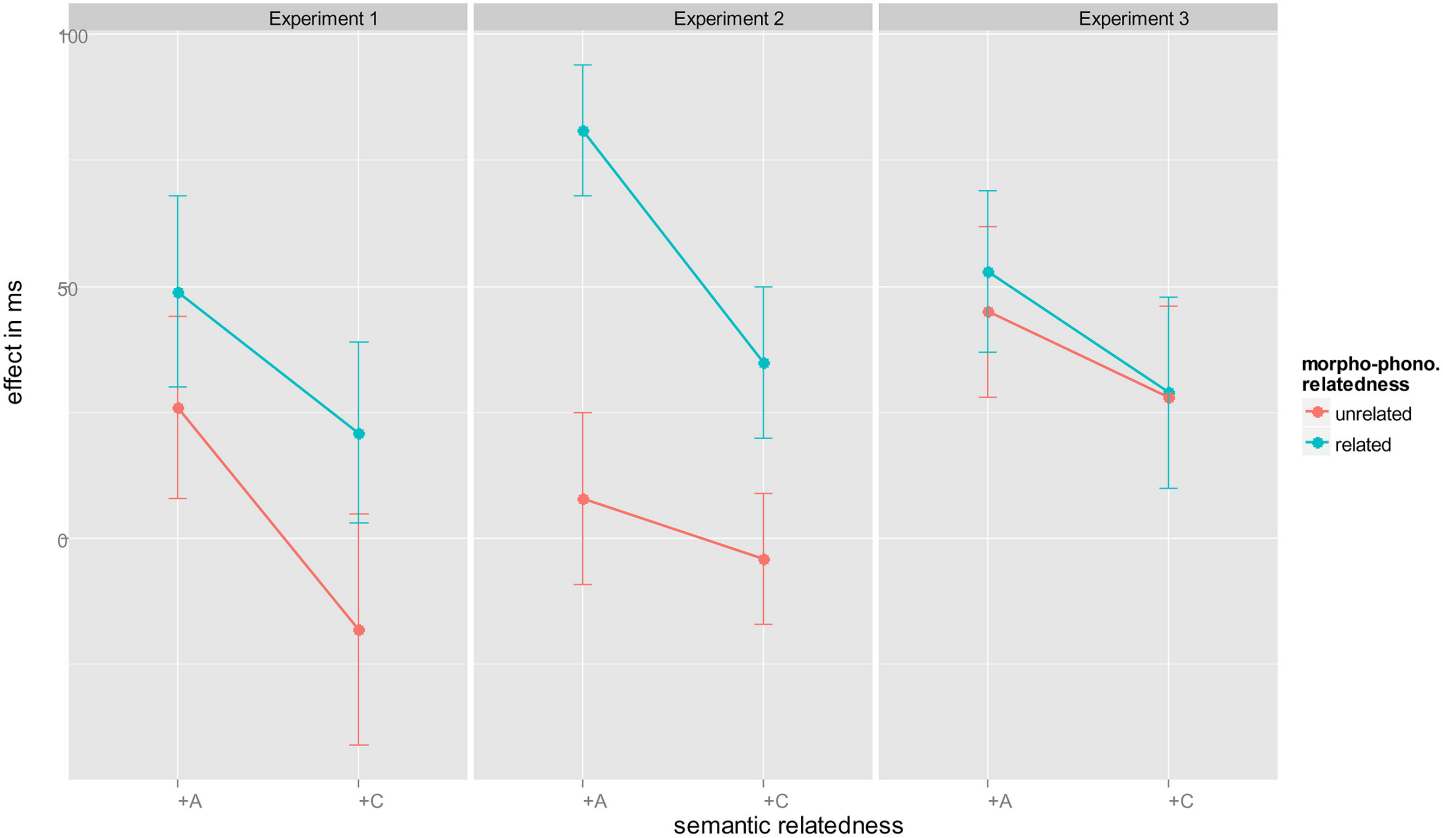
**Effects in ms [reaction times (RT) control condition – RT experimental condition] as a function of experiment, semantic, and morpho-phonological relatedness.** Experiments 1–3: +A, associatively related; +C, categorically related; +M, morpho-phonologically related; –M, morpho-phonologically unrelated. Error bars are 95% CI (see Morey, 2008).

Responses different from expected names (1.6%), disfluencies (.8%), voice-key failures (0.1%), and time-outs (1.0%) were excluded from the analyses. Responses given before cue onset were also excluded (2.4%). No item set, but two participants had to be excluded from the analyses due to missing data. Mean voice-key latencies measured from cue onset served as dependent variable^4^. (see **Table 2** for mean reaction times (RT) and standard errors; **Figure 1** displays the effects (RT control condition – RT experimental condition) per experiment). Repeated-measurement factors were Presentation (1–5) and Distractor Type (+A+M, +C+M, +A–M, +C–M, unrelated) in an initial two-ways repeated measures ANOVA. Participants named pictures faster toward the end of the experiment, as indicated by a significant linear trend for the factor Presentation [*F*_(1,37)_ = 96.469, *MSE* = 9739, *p* < 0.001, $\eta_{g}^{2}$ = 0.723]. There was no significant interaction of Distractor Type and Presentation, *F* < 1. Therefore, Presentation was dropped from further analyses. Most importantly, this analysis also yielded a significant main effect of Distractor Type [*F*_(4,148)_ = 5.983, *MSE* = 17894, *p* < 0.001, $\eta_{g}^{2}$ = 0.021]^5^.

**Table 2:** Mean picture naming latencies and standard error (in parentheses) as a function of Distractor Type and Experiment.

Experiment	Distractor Type				
	+A+M	+C+M	+A–M	+C–M	Unrelated
1	462 (25)*	490 (26)	485 (26)*	529 (27)	511 (30)
2	699 (21)*	745 (20)*	772 (23)	784 (20)	780 (21)
3	730 (24)*	754 (24)*	738 (24)*	755 (26)*	783 (28)

The asterisk (*) denotes a significant difference to the unrelated condition.

A two-ways repeated measure ANOVA with the factors Morphological Relatedness (related, unrelated) and Semantic Relatedness (associated, categorically related) using effect as dependent variable (control condition–experimental condition) yielded two significant main effects and a non-significant interaction (Morphological Relatedness: *F*_(1,37)_ = 8.024, *MSE* = 3835, *p* = 0.007, $\eta_{g}^{2}$ = 0.029; Semantic Relatedness: *F*_(1,37)_ = 13.810, *MSE* = 2966, *p* = 0.001, $\eta_{g}^{2}$ = 0.038; interaction: *F* < 1.

Mean voice key latencies of the +A+M condition were faster than those of the unrelated condition [one-sided *t*-tests: *t*_(37)_ = –3.442, *p* = 0.001] and those of the associative condition without morpho-phonological overlap, +A–M [*t*_(37)_ = –2.517, *p* = 0.016]. There was a trend toward significance when comparing the +A+M mean voice key latencies with those of the +C+M condition [*t*_(37)_ = –1.585, *p* = 0.061]. Notice that we did not correct these and all following *post hoc* tests for multiple comparisons. Mean picture naming latencies in the category distractor condition +C–M were numerically longer but did not differ significantly from those in the unrelated condition [two-sided *t*-test: *t*_(37)_ = 1.045, *p* = 0.303]. Thus, as in previous research, same-category members showed no facilitation, but also did not reliably interfere with picture naming in a picture–picture setting (cf. Glaser and Glaser, 1989; La Heij et al., 2003). Note that the main effect of semantic relatedness was significant, showing that an associative relation between distractors and target induced facilitation (37 ms) but a categorical relation did not (2 ms).

Fixations and dwell-time were measured from the onset of the pictures, with the help of the EyeLink Data Viewer program. Dwell-time was defined as the summation of the duration of all fixations on an interest area. Fixations reflect whether a specific item was fixated at all, from picture onset until reaction or trial end.

The eye-tracking data showed that participants fixated only one of the displayed objects in 36.6% of the trials (target: 29.1%, one distractor: 7.5%). Two objects were fixated in 33.9% of the trials (target and one distractor: 31.9%, both distractors: 1.7%). All three objects were looked at in 10.9% of the trials. All other fixations (19.0%) fell outside the objects (see **Table 3** for an overview). The number of gazes shows that participants looked at the target object most often, which does not come as a surprise. As has been known for a long time, fixations – as a measure of overt attention – are not needed for the correct perception of objects or scenes (Fei-Fei et al., 2005). Evidently, targets can be and were named correctly without overt attention, and it is thus very likely distractors can also exert an influence on target naming without overt attention. Thus, overlapping stimulus configurations, as in the variant of Morsella and Miozzo (2002) are not mandatory for obtaining voice-onset latency effects of distractors. However, the visual angle and presentation time used here allow covert attention shifts. Two ANOVAs, one with first fixation onset on the target, the other with dwell time on the target as dependent variable and Distractor Type as factor showed no significant effects (*F* < 1).

**Table 3:** Percentage gazes broken down by condition and fixated object for Experiments 1–3.

		Distractor condition				
Experiment	Fixated object	+A+M	+C+M	+A–M	+C–M	Unrelated
1						
	Target	6.9	5.1	5.7	5.8	5.6
	Distractor(s)	1.5	1.9	2.1	1.9	1.8
	Target and distractor(s)	7.8	9.1	8.7	8.4	8.7
	Nothing	4.0	3.9	3.4	3.7	4.0
2						
	Target	11.0	9.7	9.9	9.5	8.7
	Distractor(s)	0.6	0.7	0.6	0.5	0.5
	Target and distractor(s)	6.1	7.6	8.1	8.3	8.7
	Nothing	2.4	1.9	1.4	1.7	2.1
3						
	Target	12.2	11.2	10.3	11.4	10.6
	Distractor(s)	0.2	0.1	0.1	0.1	0.3
	Target and distractor(s)	5.6	6.6	7.1	6.3	6.6
	Nothing	1.3	1.1	1.6	0.8	1.3

### Discussion

To summarize, Experiment 1, with 600 ms time before the target was signaled, revealed both semantic effects (positive and null) as well as facilitation by shared morpho-phonological information with distractor pictures. Distractor pictures that were associatively related to the target picture clearly speeded target naming. Overall, categorically related distractor pictures showed no effect (2 ms). The large and reliable difference between the two semantic conditions, evident in the main effect of semantic relation, with 37 ms facilitation due to associatively related distractors but no effect for categorical distractors (2 ms), is in fact evidence for an impact of lexical competition on conceptually induced facilitation.

This modulation of conceptual/semantic facilitation by lexical competition fits with discrete models, but not with fully cascading models (Caramazza, 1997), nor with the response-buffer explanation of interference caused by word distractors (Mahon et al., 2007). The main effect of morpho-phonological relatedness, with 33 ms facilitation when morphological relatedness is present but no effect (4 ms) without such overlap, clearly indicates the presence of word-form information of distractor pictures. This replicates the word-form effects with overlapping, colored picture presentation (Morsella and Miozzo, 2002), and provides evidence for full cascading within the language production system.

**FIGURE 2:**
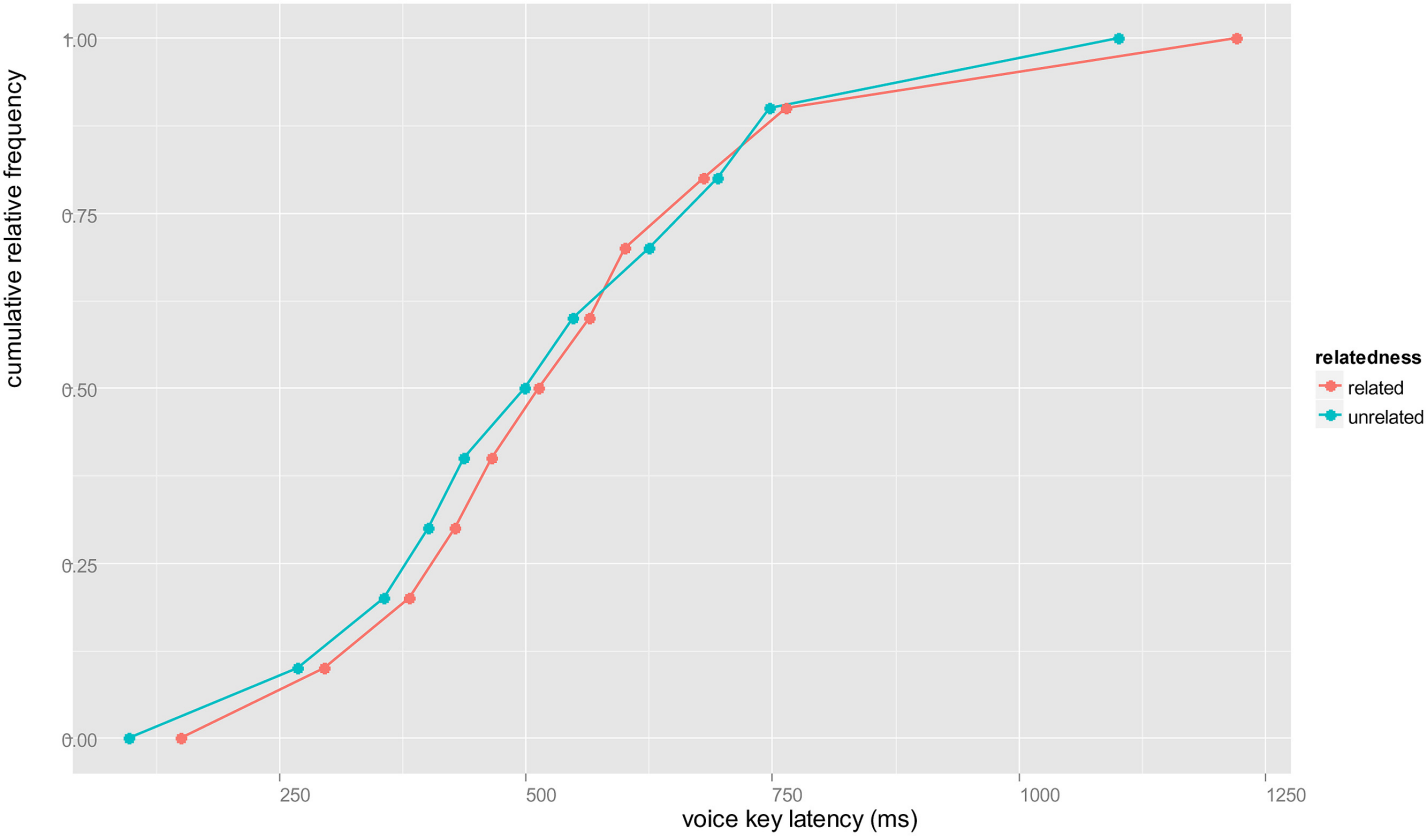
**Vincentized cumulative distribution curves for voice key latencies of the unrelated and +C–M condition.** RTs of the unrelated condition are predominantly above the RTs of the +C–M condition. An intersection of the curves over a longer period would indicate that interference develops at a certain latency range (Ratcliff, 1979; Roelofs, 2008b).

To our knowledge, there are no picture–picture studies with associative relations between distractors and target. Our participants named target pictures faster in the presence of associatively related distractors. This replicates results from PWI studies (e.g., Bölte et al., 2003, 2005; Costa et al., 2005). Whereas semantic facilitation can be explained by activation at the non-lexical, conceptual level (see also La Heij et al., 2003), the fact that such semantic effects disappear when distractors and targets are from the same semantic category clearly indicates lexical involvement. Unlike others, we obtained no reliable interference relative to the unrelated condition. The closest comparison is a study by Humphreys et al. (1995), who also used a post-cue picture–picture procedure and observed semantic inference for categorically related pairs (e.g., horse–tiger). One difference between our study and Humphreys et al. (1995) is that naming responses were very slow, nearly twice as slow as ours. This suggests that interference might develop over time, but visual inspection of our data does not support this (see **Figure 2**), as there is no indication of interference at longer RTs.

Let us turn now to the interpretation of the “null effects” for categorical distractors. One argument could be that the distractor pictures never entered the lexical system to start with. But if distractors are not lexicalized, no effects of morpho-phonological relatedness should have been observed. In the absence of associative distractors, it would have been difficult to interpret the null effect, but compared to the clear facilitation for associative stimuli, the null effect seems to indicate that interference occurred, but was canceled out by facilitation due to semantic similarity. Note that according to the pretest, associative, and categorical stimuli were equally related to their targets. The combination of facilitatory conceptual effects, both for categorical and associative distractors, with an inhibitory lexical effect for categorical distractors only fits well with the idea of lexical competition implemented in the model proposed by Levelt et al. (1999). Semantic competition due to picture distractors is not predicted by the cascading model by Caramazza (1997), nor is it compatible with the post-lexical explanation of semantic interference that was devised for effects of word distractors (Mahon et al., 2007).

The type of semantic relation and the position of morphological overlap between distractors and target are naturally confounded. Associatively related distractors (e.g., *garden gnome*) overlap with the target name (e.g., *garden chair*) in their onset, sharing their modifier, while categorically related distractors overlap with the target in head position (e.g., *rocking chair*). There are no left-headed compounds in German that would allow separating overlap and semantic relatedness. Given that all three picture names started the same (e.g., *garden gnome, garden chair, garden fence*), participants could have prepared at least the modifier, in trials with associated stimuli, before even knowing which one was the target. Note, however, that this was not possible for the +A–M condition, which also showed semantic facilitation. Nevertheless, some additional processing advantage in the +A+M condition might result from phonological preparation – which still constitutes a down-stream lexical effect of word-form access and phonological encoding.

Given the SOA of 600 ms, it is quite possible that our participants started the lexical encoding of one or more pictures before the cue appeared. Although in discrete models, a parallel phonological encoding should not happen even in those situations, Experiment 2 was designed to minimize such preparation effects, by reducing the cue onset time to 200 ms.

## Experiment 2: Cue Onset 200 ms

We reduced the SOA between the onset of the three pictures and the cue from 600 to 200 ms. A shorter cue-onset asynchrony provides less time for lexical activation of all pictures, and thus less time for an impact of lexical competition and of word-form similarity. Hence, a phonological preparation effect that might help target naming in cases of onset overlap (as with the associatively related stimuli) could be reduced. As a consequence, overall positive semantic (associative and categorical) effects, if present, might become more pronounced.

### Method

#### Participants

Forty participants selected from the same population as before were tested. None had participated in Experiment 1 or in the pretests. They received the same compensations as the participants of Experiment 1. All had normal or corrected-to-normal vision and were native speakers of German.

#### Procedure

The same material and apparatus as in Experiment 1 was used. The only difference to the previous experiment was that the cue signaling the target appeared 200 ms after the onset of the three pictures, instead of 600 ms. All other aspects of the procedure remained the same.

### Results

Responses different from expected names (2.1%), disfluencies (0.5%), voice-key failures (0.6%), time-outs (2.6%) and reactions before cue onset (0.2%) were excluded from the analyses. No item set or participant had to be excluded from the analyses. **Table 2** lists mean RTs and standard errors as a function of Distractor Condition. One difference to Experiment 1 is obvious at first sight: latencies are much longer overall.

Voice-key latencies measured from cue onset were averaged over participants and submitted to separate ANOVAs. We first analyzed the results with Presentation (1–5) and Distractor Type (+A+M, +C+M, +A-M, +C–M, unrelated) as factors. A significant linear trend for the factor Presentation indicated that participants named pictures faster toward the end of the experiment [*F*_(1,39)_ = 105.700, *MSE* = 27091, *p* < 0.001, $\eta_{p}^{2}$ = 0.730]. There was no significant interaction between Distractor Type and Presentation, *F* < 1. Therefore, the remaining analyses are presented collapsed across this factor. The main effect of Distractor Type was significant [*F*_(4,156)_ = 23.546, *MSE* = 10634, *p* < 0.001, $\eta_{g}^{2}$ = 0.044].

In a two-ways repeated measures ANOVA (Morphological Relatedness: related vs. unrelated; Semantic Relatedness: associatively vs. categorical) using effect as dependent variable, there were significant main effects of Morphological Relatedness [*F*_(1,39)_ = 52.617, *MSE* = 2384, *p* < 0.001, $\eta_{g}^{2}$ = 0.157] and of Semantic Relatedness [*F*_(1,39)_ = 17.935, *MSE* = 1885, *p* < 0.001, $\eta_{g}^{2}$ = 0.048]. Overall, morphologically related distractors yielded facilitation (58 ms), but morphologically unrelated ones did not (–2 ms). Moreover, effects were larger for associatively related (44 ms) than for categorically related distractors (19 ms). The interaction was also significant in [*F*_(1,39)_ = 6.810, *MSE* = 2048, *p* = 0.013, $\eta_{g}^{2}$ = 0.020]. The interaction was due to the fact that the difference between +A–M and +C–M was only 12 ms, while the difference between +A+M and +C+M was 46 ms. When distractors were morphologically related to their target, associatively related distractors facilitated naming responses more than categorically related ones. When there was no morphological relation, associatively and categorically related distractors were equally ineffective.

Mean voice key latencies were faster of both morpho-phonological conditions relative to the unrelated condition: +A+M [*t*_(39)_ = –7.303, *p* < 0.001] and +C+M, [*t*_(39)_ = –3.391, *p* = 0.001]. Furthermore, there was a significant difference between these two [*t*_(39)_ = –4.789, *p* < 0.001]. Associatively related distractors without morpho-phonological overlap did not differ from the unrelated condition, +A–M [*t*_(39)_ = –0.654, *p* = 0.259], and the same was true for category members without morpho-phonological overlap +C–M [*t*_(39)_ = 0.568, *p* = 0.287].

We had hypothesized that the facilitatory effect of the +A+M condition could be due to a phonological preparation effect. In Experiment 1 participants had approximately 600 ms to prepare the modifier of the compound as first part of naming the target picture. The shortened cue onset (SOA) of Experiment 2 should reduce the influence of this hypothesized effect.

We tested this in an ANOVA with the data from both experiments/SOAs. Given that the overall latencies were quite different, we first *z*-transformed the RT for each SOA (600, 200), and used the effect of the morpho-phonologically related conditions (unrelated condition – related; +A+M, +C+M, respectively) as dependent variable. The ANOVA included the factors SOA (600, 200) and Semantic Relatedness (associated, categorically related). Semantic relatedness did matter [*F*_(1,78)_ = 16.053, *MSE* = 0.241, *p* < 0.001, $\eta_{g}^{2}$ = 0.046]. Neither SOA [*F*_(1,78)_ = 1.201, *MSE* = 0.782, *p* = 0.277] nor the interaction [*F*_(1,78)_ = 2.083, *MSE* = 0.241, *p* = 0.153] were significant. Thus, irrespective of SOA, given morpho-phonological overlap between distractor and target pictures, associatively related distractor pictures induced more facilitation (63 ms) than categorically related ones (28 ms). Note again that no effects were found in Experiment 2 in the absence of morpho-phonological overlap.

The eye-tracking data showed that participants fixated only one object in 51.3% of the trials (target: 48.7%, one distractor: 2.6%). Two objects were fixated in 33.9% (target and one distractor: 33.3%, both distractors: 0.3%). All three objects were looked at in 5.3% of the trials. All other fixations (9.6%) fell outside the objects (see **Table 3** for an overview). The number of gazes shows that participants looked at the target object even more often than in Experiment 1.

An ANOVA with first-fixation onset as dependent variable showed a significant effect for Distractor Type [*F*_(4,152)_ = 12.392, *MSE* = 822, *p* ≤ 0.001, $\eta_{g}^{2}$ = 0.136]^6^. To further investigate this difference, we ran a two-ways repeated measures ANOVA, with Morphological Relatedness (related, unrelated) and Semantic Relatedness (associatively, categorically related) as factors. Targets attracted faster fixation onsets in the presence of morpho-phonologically related distractors than with unrelated ones [*F*_(1,38)_ = 33.566, *MSE* = 919, *p* ≤ 0.001, $\eta_{g}^{2}$ = 0.136+M: 422 ms; –M: 450 ms]. Overall, targets were looked at faster in the presence of associatively related than with categorically related distractors [*F*_(1,38)_ = 5.094, *MSE* = 573, *p* = 0.03, $\eta_{g}^{2}$ = 0.015; +A: 432 ms, +C: 441 ms]. A marginally significant interaction qualified the main effects [*F*_(1,38)_ = 3.675, *MSE* = 822, *p* = 0.063, $\eta_{g}^{2}$ = 0.015; +A+M: 414 ms, +C+M: 431 ms, +A–M: 451 ms, +C–M: 450 ms]. The interaction showed that the faster fixations to targets in the presence of associatively related distractors only held when the picture names were morpho-phonologically related, not in the absence of morphological relatedness.

The one-way repeated measure ANOVA with the within factor Distractor Type on dwell-time was also significant [*F*_(4,152)_ = 2.477, *MSE* = 6475, *p* = 0.047, $\eta_{g}^{2}$ = 0.005]. Again, we followed this analysis by the same two-ways repeated measures ANOVA as reported before. Only the factor Semantic Relatedness was significant [*F*_(1,38)_ = 10.326, *MSE* = 3876, *p* = 0.003, $\eta_{g}^{2}$ = 0.013]. Associatively related distractors induced shorter dwell times on the targets than categorically related ones. The factor Morphological Relatedness and the interaction were not significant [*F*_(1,38)_ ≤ 1.424, *p* ≤ 0.240].

### Discussion

Experiment 2 showed similar effects as Experiment 1. Morpho-phonological relatedness between target and distractor pictures (+A+M, +C+M) facilitated picture naming, relative to an unrelated baseline and to morpho-phonologically unrelated distractors. The observed effects do not seem to originate from preparation of the first morpheme shared by distractors and target in the +Ä+M condition, as corroborated by the lack of interaction in the analysis on the data from both SOAs. Different from Experiment 1, associatively related distractors did not facilitate target picture naming when they were morpho-phonologically unrelated. The shortened SOA and/or the absence of a morpho-phonological association may have prevented the full build-up of positive associative as well as negative categorical effects, but apparently did not prevent access to lexical information for the pictures. Numerically, effects of morpho-phonological similarity between the names of distractor and target pictures were even stronger than in Experiment 1. Thus, manipulating SOA seems to differentiate between the strength of semantic-conceptual (associative and categorical) and lexical (presence or absence of morpho-phonological influences) effects. Importantly, as in Experiment 1, the data provide evidence for full cascading to the word-form level, and the substantial difference between effects of associatively related (44 ms) and categorically related distractors (19 ms) at least is compatible with interference due to lexical competition.

In Experiment 3, we changed the way in which it was signaled which picture was the target for naming. Glaser and Glaser (1989) asked their participants to name the first (or the second) picture that appeared on the screen (see also La Heij et al., 2003). We adapted this procedure and signaled the target by a later onset. We wanted to give the distractor pictures a head start, attracting attention by means of their visual onset, to allow for a full impact of conceptual/semantic effects (we argued that it is impossible to avoid semantic processing of visual stimuli). Given that it is clear that the distractor pictures always come first, they may well not be processed lexically at all, because they should not be named. If this holds, there should be no impact of morpho-phonological relatedness, or of lexical competition. Thus, with this presentation manipulation, we investigated whether lexical processing of stimuli that do not have to be named is mandatory, and if so, up to which level. Another important motivation for Experiment 3 is to assess potential differences in the strength of the semantic relation between target and distractor pictures in the four conditions. Although the mean semantic-relatedness judgments (see **Table 1**) did not differ, the small differences between the means could have an impact when online priming effects are concerned. If the data from Experiment 3 show pure semantic effects, without any lexical competition or morpho-phonological involvement, the priming by the four distractor conditions would be purely conceptual and could be compared directly.

## Experiment 3: Target 200 ms after Distractors

In this experiment, we altered the way the target picture was signaled. In Experiments 1 and 2, we used an arrow that appeared some time after the simultaneous onset of all three pictures, to indicate the target picture. In Experiment 3, the target picture appeared 200 ms after the onset of the distractor pictures. This provides some time for the processing of the distractors, and gives them a head start. This SOA also roughly corresponds to SOAs used in PWI experiments to evoke semantic effects – but note that the processing flow differs for pictures and words. Most importantly, we reasoned that this timing would give rise to positive conceptual-semantic effects, perhaps to competition effects, but not to word-form effects. Positive semantic effects of associative and categorical distractors should be evident because the earlier distractor onset allows activating the relevant conceptual network (Abdel Rahman and Melinger, 2009).

### Method

#### Participants

Twenty participants selected from the same population as before were tested in this experiment. None had participated in Experiments 1 and 2.

#### Procedure

The same material and apparatus as in Experiments 1 and 2 was used. The difference to the previous experiment was that the target picture appeared 200 ms after distractor-picture onset. Furthermore, we changed the filler conditions. In 12 of the 24 fillers, distractor pictures were replaced by pictures with morpho-phonological overlap either in the first (6) or second constituent (6) of the other distractor picture. Note that the target picture was never morpho-phonologically related to these distractor pictures. However, given the different timing of distractors and target, we wanted to counteract strategic processing induced by the distractor pair (i.e., whenever there is morpho-phonological overlap in the first or second constituent of the distractor pictures, the target picture shares this constituent). The target pictures in the filler condition also had different distractor pictures in each of the five presentations. Additionally, the distractor pictures without morpho-phonological overlap in the filler condition were randomized further within themselves. All other aspects of the procedure remained the same.

### Results

Responses different from expected names (1%), disfluencies (1.3%), voice key failures (0.4%), time-outs (1.8%) and reaction before target onset (0.1%) were excluded from the analyses. No item set or participant was excluded from the analyses. Voice-key latencies measured from target picture onset were averaged over participants and submitted to an ANOVA. **Table 2** lists RT and standard errors as a function of Distractor Type. Participants named pictures faster toward the end of the experiment, indicated by a significant linear trend [*F*_(1,19)_ = 23.823, *MSE* = 26969, *p* = 0.001, $\eta_{g}^{2}$ = 0.556]. There was no significant interaction between Distractor Type and Presentation (*F* < 1). Therefore, the remaining analyses were collapsed across this factor. There was a main effect for the factor Distractor Type [*F*_(4,76)_ = 5.529, *MSE* = 1454, *p* = 0.001, $\eta_{g}^{2}$ = 0.030].

Using effect as dependent variable, the main effect of Morphological Relatedness *F* < 1) was not significant nor the interaction were significant in the two-ways repeated measures ANOVA. The main effect of Semantic Relatedness was marginally significant [*F*_(1,19)_ = 3.154, *MSE* = 2240, *p* = 0.092, $\eta_{g}^{2}$ = 0.030].

The significant main effect for the factor Distractor Type was further analyzed using paired one-sided *t*-tests and averaged voice key latencies as dependent variable. Participants were faster in naming the target picture relative to the unrelated condition in the associatively and morpho-phonologically related condition, +A+M [*t*_(19)_ = –4.067, *p* < 0.001] and in the categorically and morpho-phonologically related condition, +C+M [*t*_(19)_ = –2.465, *p* = 0.012]. Facilitation was also significant for both conditions without morphological overlap: +A–M [*t*_(19)_ = –3.644, *p* = 0.001], +C–M [*t*_(19)_ = –2.535, *p* = 0.010].

The eye-tracking data analyzed after target onset showed that participants fixated one object in 57.1% of the trials (target: 56.3%, one distractor: 0.8%). Two objects were fixated in 32.2% (target and one distractor: 22.4%, both distractors: 0.1%). All three objects were looked at in 5.0% of the trials. All other fixations (6.1%) fell outside the objects (see **Table 3** for an overview). Interestingly, the eye-tracking data before target onset demonstrated that both distractors were only fixated in 0.1% of the cases, whereas one of the two distractors was looked at in 36.6%. All other fixations fell outside of the objects (63.3%). First fixation onsets as well as dwell-times did not differ from each other (*F* < 1).

### Discussion

Consistent with our expectation, we observe facilitation in all conditions relative to the unrelated baseline: for both associatively (+A+M = 53 ms, +A–M = 45 ms) and both categorically related distractors (+C+M = 29 ms, +C–M = 28 ms), irrespective of morpho-phonological relatedness. Different from Experiments 1 and 2, category members reliably facilitated picture naming in a picture–picture setting. Thus, we replicate earlier findings that an onset manipulation gives rise to semantic effects (see Glaser and Glaser, 1989; La Heij et al., 2003, for inhibitory effects).

The results from Experiment 3 indicate that distractor pictures were not processed lexically. The effects of the two semantic conditions are statistically the same: both speed up target naming, most probably due to semantic priming through spreading of activation at the conceptual level. There is no evidence for lexical competition with distractor pictures from the same semantic category; both conditions induce significant semantic priming. The effect in the associative conditions is numerically larger (20 ms) than in the categorical conditions, but note that the main effect of Semantic Relatedness failed significance. The numerical difference might reflect the somewhat larger semantic relatedness scores from the pretest (mean semantic relatedness rating: associatively related: 3.65 vs. categorically related: 3.20). In the absence of lexical competition effects, it is not surprising that distractors are not processed all the way down to the word-form level. Otherwise, we should have observed an additional effect of morpho-phonological overlap, which has proven to be facilitatory over a wide range of material and tasks (Roelofs and Baayen, 2002; Gumnior et al., 2006; Lüttmann et al., 2011a,b), as well as in Experiments 1 and 2.

## General Discussion

This study aimed to test two competing types of model of speech production: the two-step discrete serial model (Levelt et al., 1999) and models that allow full cascading of lexical information for multiple concepts, all the way down to word-form and phonological levels (Caramazza, 1997; Peterson and Savoy, 1998). An additional aim was to test differing explanations for semantic interference from word distractors on target processing, which can be ideally tested with picture distractors. We found encoding of distractor names up to the form level, which supports cascading rather than serial models. We also observed interference from distractors on target naming which is not predicted by a post-lexical response buffer explanation (Mahon et al., 2007).

To test the predictions of the competing models, we focused on potential facilitation and inhibition effects of different types of semantic similarity, and on the morpho-phonological relation between distractor and target pictures in picture naming. We manipulated semantic (categorical or associative) relatedness, crossed with morpho-phonological overlap (present or absent). We did so to assess the level up to which distractor pictures, whose names are *not* produced, are lexically encoded. Furthermore, the distractor pictures and the target picture appeared simultaneously or staggered, with the distractor pictures preceding the target picture. In case of simultaneous presentation, the cue signaling the target appeared at different moments in time. The target and cue onset manipulations served to gain insight in the temporal aspects of distractor processing.

Our versions of the picture–picture paradigm show that overlapping pictures and color as signal, as used by Morsella and Miozzo (2002), are not necessary to evoke effects. Varying temporal onsets of distractor and target, or signaling the target by means of a cue, are both effective manipulations and reveal semantic effects that were not observed with the color manipulation (see also Glaser and Glaser, 1989; La Heij et al., 2003). Moreover, it was not necessary to focus attention to the distractor pictures by spatial cueing, as done by Jescheniak et al. (2014) to induce lexical competition by distractor pictures. We simply presented the distractors first, and their onset was enough to attract attention and induce semantic processing. In addition, the cue technique induced form effects from distractors pictures that shared a morpheme with the target picture (Experiments 1 and 2).

Before moving to the effects observed and their relevance for the predictions made by discrete and cascaded models, we discuss the eye-tracking data. Effects of the experimental manipulations in the eye-tracking data emerged only in Experiment 2, where morphologically related distractors accelerated first-fixation onsets, and associatively related distractors were fixated faster than categorically related ones. Dwell times partly mirrored the pattern of the first-fixation onsets, but without effects of morphological relatedness. One might conclude that the implemented experimental situations were not demanding enough to affect eye-movements, although they effectively affected word-production processes. The role of eye-movements is less well understood in word production than in reading (Rayner, 2009). Meyer et al. (1998) suggested that eye-movements reflect the timing of word-production processes in multi-word utterances. However, this is not mandatory, because speakers can deviate from the observed coupling of eye-movements and word production when the task is easy (Meyer et al., 2012).

In our experiments, eye movements were tracked to investigate whether target fixations are mandatory for accurate naming, and whether fixations on distractor pictures are necessary for effects to emerge. The answer to both seems to be no. In Experiment 1, in approximately 28% of the cases the target was not fixated at all, but naming was very accurate indeed (~94%). Moreover, distractors were rarely fixated alone (9% compared to targets alone: 29%). In about half of the trials (48%) no distractor was looked at but we still get clear effects of distractors on target naming. Overt attentional shifts to distractors, as indicated by eye-movements, are thus not required for their lexical encoding. This replicates our findings with scene stimuli (Dobel et al., 2007).

### Semantic Relatedness

Discrete two-step models implement two lexical levels, lemmas that code syntactic information, and lexemes or word-forms that code morphological and phonological information. Such models allow for the activation of multiple lemmas at the first level, but – with few exceptions – not of multiple word forms.

In two experiments (Experiments 1 and 3) associatively related distractor pictures accelerated target picture naming, even without morpho-phonological similarity. Thus, related concepts such as *lawn mower* and *swimming pool* facilitate the naming of the picture of a *garden chair*. In Experiment 2, with a shorter target-cue onset, facilitation emerged only when distractors (*garden hose*; *rocking chair*) and target (*garden chair*) shared a morpheme. If pictures all belong to – very loosely speaking – the same semantic field, their concepts seem to activate each other, which speeds up conceptual processing and target picture naming.

When the target picture was signaled by means of a cue, categorically related distractors induced neither facilitation nor interference, relative to the unrelated baseline. But facilitation due to categorically related distractors (e.g., *kitchen table* and *shoe rack*) was only observed when distractor pictures and target picture appeared at different moments in time (Experiment 3). This seems at odds with results by Glaser and Glaser (1989) and La Heij et al. (2003, Experiment 1). Glaser and Glaser (1989) observed interference and argued that this is because distractor and target activate closely related semantic representations. La Heij et al. (2003) proposed that effects observed by Glaser and Glaser (1989) were due to the erroneous selection of distractors as target. Moreover, Glaser and Glaser (1989) used just nine pictures as target and context pictures. When La Heij et al. (2003) reduced distractor-presentation duration from 300 to 50 ms and increased the number of target pictures from 9 to 40, they observed facilitation. We used longer distractor presentation durations than La Heij et al. (2003), had a smaller number of target pictures, but observed facilitation nonetheless (Experiment 3). Thus, it is most likely that neither the number of target pictures nor the distraction presentation duration is the crucial manipulation. Observing semantic facilitation, or interference, rather depends on the ease of target identification. When the target is clearly signaled and distractor pictures are not used as targets in the experiment, effects are facilitatory. In these cases, distractors do not seem to enter the lexical system (as in Experiment 3). If there is (temporal) uncertainty as to which picture is going to be the target, lexical access is initiated for all pictured concepts, rendering lexical selection of the target more difficult when the distractors come from the same semantic category (Glaser and Glaser, 1989; La Heij et al., 2003). This is what we observed in Experiments 1 and 2. We feel that the situation of uncertainty, which concept to express, which environmental stimulus to name, is rule rather than exception during speaking. This is clearly reflected in the fact that all models of speech production adhere to the activation of multiple conceptual representations, and all models allow these non-linguistic representations to activate linguistic ones. As such, certainty as to which pictures are targets for naming and which not (Experiment 3) is the exception, rather than the rule.

A next question is, how “categorical” facilitation (Experiment 3) occurs. As our data show: in a similar manner to associative facilitation. Related concepts activate each other, speeding up target processing at conceptual and subsequent stages, even all the way down to the vocal response. The result challenges the assumptions made by Levelt and Colleagues Roelofs (1992) and Levelt et al. (1999), who claim that conceptual activation always results in the activation of multiple lemmas, which compete for selection. The data from Experiment 3 show that categorically related distractor pictures did activate their conceptual-semantic information, which apparently was not fed forward into the lexical stratum, because we observed no interference. Our data suggest that lexical processing of activated concepts is not mandatory. When there is no uncertainty as to which picture has to be named, the distractors, although activated at the conceptual level, do not enter the lexical system. This in fact also fits with Roelofs (2008a), who argued that task demands determine the presence and direction of semantic effects. When target selection is easy, facilitation occurs, while in case of a difficult selection, inhibition is observed.

When there is uncertainty as to which target should be named (Experiments 1 and 2), we do indeed observe inhibition from distractor pictures that share their semantic category with the target – relative to associatively related distractors. This is evidence for lexical competition and selection (Roelofs, 1992). Lexical selection by competition is not implemented in full cascading models such as the one proposed by Caramazza (1997), Finkbeiner and Caramazza (2006), and Mahon et al. (2007). Consequently, they proposed a different locus for the interference from categorically related distractor words regularly observed in picture naming: the post-lexical response buffer. Given that our interference comes from pictures, not from words, this refutes the response-buffer explanation, because only word distractors can cause havoc in the response buffer, the locus of the interference in their model. (Mahon et al., 2007).

### Morphological Relatedness

In Experiments 1 and 2, the morphological relation between distractors and target modulated the effects obtained for semantic relatedness. This finding shows that distractor pictures were processed all the way “down” to the form level. These data do not agree with results by Meyer et al. (1998), who found separate and sequential processing, from meaning to phonology, for two simultaneously displayed pictures that both had to be named. But note that this is a special situation (Meyer et al., 2012). Importantly, our results argue against views that allow no multiple lexical activation at all (Bloem and La Heij, 2003), and against partially cascaded models that only allow a “limited” flow of activation from conceptual to form representations (Levelt et al., 1991, 1999; Roelofs, 1992, 2003).

According to all models, the conceptual level is “blind” to the morpho-phonological structure of the word belonging to a concept. To observe morpho-phonological facilitation, all concepts must have been processed to a level at which this information is represented, and this must be the lexeme or word-form level. Obviously, the semantic cohort of the target that is set up upon lexical access will incorporate morphologically related words, given that these often are semantically related. However, it has been shown in a variety of tasks that semantic and morphological effects in speech production reflect processing at different levels, and that morphological similarity without semantic relatedness (as with the “hummingbird” and the “jailbird”) is (almost) as effective as with semantic similarity (“hummingbird” and “blackbird”; e.g., Dohmes et al., 2004; Koester and Schiller, 2008, 2011; Lüttmann et al., 2011b).

Taken together, the morpho-phonological effects fit with fully cascaded models of speech production (Stemberger, 1985; Dell, 1986; Caramazza, 1997; Peterson and Savoy, 1998), in which it is assumed that activation flows continuously from high levels to lower levels. Note that the evidence for competitive lexical selection, based on the nature of the semantic relation between distractor and target pictures, is a challenge to some of these models. The fact that the influence of morpho-phonological relatedness varies from experiment to experiment, and is even absent when there is no uncertainty about the target, suggests that the flow of information depends on specific characteristics of the speaking situation (Roelofs, 2008a). Roelofs and Piai (2011) as well as O’Séaghdha and Frazer (2014) argue that the degree of phonological activation depends on the available attentional capacity. Thus, it is an assignment for any model of speech production to adjust the claims about their basic structure to the requirements of the task, the speaking situation and to the amount of attention paid to stimuli in the environment.

## Ethical Statement

This study was carried out in accordance with the recommendations of ethical guidelines of the Institute for Psychology, Westfälische Wilhelms-Universität, Münster, Germany, with written informed consent from all participants. All participants gave written informed consent in accordance with the Declaration of Helsinki.

## Conflict of Interest Statement

The reviewer, Dr. Markus Damian, declares that despite having collaborated with the author Dr. Jens Bölte, the review process was handled objectively. The authors declare that the research was conducted in the absence of any commercial or financial relationships that could be construed as a potential conflict of interest.

## Supplementary Material

The Supplementary Material for this article can be found online at: http://journal.frontiersin.org/article/10.3389/fpsyg.2015.01540

## Footnotes

1 Roelofs (2008a) formulated an exception, allowing for the incidental cascading to word-form information. One and the same concept may activate multiple word forms, as is the case for near synonyms (e.g., *sofa* and *couch*).

2 German compounds are written without spaces.

3 We use the term morpho-phonological overlap to signal that the target constituent overlaps phonologically with the distractor constituent. The phonological overlap constitutes at the same time a free morpheme. Morpho-phonological overlap is different from pure phonological overlap (Roelofs and Baayen, 2002; Zwitserlood et al., 2002).

4 We do not report F2-analyses in this study because targets were repeated over conditions and not nested under conditions (Clark, 1973; Raaijmakers et al., 1999). Linear mixed effects models that have been suggested as alternative to F1 and F2-analyses converged only without random slopes. The LME-analyses corroborated the reported results, but we do not report them here because we fell that the low number of trials per condition renders questionable the results of such analyses (Barr et al., 2013).

5 We used SPPS to compute $\eta_{p}^{2}$ and a spreadsheet provided by Lakens (2013) to compute $\eta_{g}^{2}$ine-formula>.

6 One participant was excluded due to the number of outliers.

## References

[B1] Abdel Rahman R. Melinger A. (2009). Semantic context effects in language production: a swinging lexical network proposal and a review. *Lang. Cogn. Process.* 24 713–734. 10.1080/01690960802597250

[B2] Alario F. Segui J. Ferrand L. (2000). Semantic and associative priming in picture naming. *Q. J. Exp. Psychol. A* 53 741–764. 10.1080/027249800410535 10994228

[B3] Aristei S. Zwitserlood P. Abdel Rahman R. (2012). Picture-induced semantic interference reflects lexical competition during object naming. *Front. Psychol.* 3:28 10.3389/fpsyg.2012.00028. PMC3281281 22363304

[B4] Baayen R. H. Piepenbrock R. Van Rijn H. (1993). *The CELEX Lexical Database.* Philadelphia, PA: CD-ROM.

[B5] Barr D. J. Levy R. Scheepers C. Tily H. J. (2013). Random effects structure for confirmatory hypothesis testing: keep it maximal. *J. Mem. Lang.* 68 255–278. 10.1016/j.jml.2012.11.001 PMC3881361 24403724

[B6] Belke E. (2005). Lexical access revisited: evidence from picture-pseudoword interference. *Eur. J. Cogn. Psychol.* 17 117–150. 10.1080/09541440340000448

[B7] Bloem I. La Heij W. (2003). Semantic facilitation and semantic interference in word translation: implications for models of lexical access in language production. *J. Mem. Lang.* 48 468–488. 10.1016/S0749-596X(02)00503-X

[B8] Bölte J. Böhl A. Dobel C. Zwitserlood P. (2009). Effects of referential ambiguity, time constraints and addressee orientation on the production of morphologically complex words. *J. Cogn. Psychol.* 21 1166–1199. 10.1080/09541440902719025

[B9] Bölte J. Jorschick A. Zwitserlood P. (2003). “Reading yellow speeds up naming a picture of a banana: facilitation and inhibition in picture-word interference,” in *Proceedings European Cognitive Science Conference*, eds Schmalhofer F. Young R. (Mawah, NJ: Lawrence Erlbaum Associates Inc.), 55–60.

[B10] Bölte J. Jorschick A. Zwitserlood P. (2005). Categorical inhibition and associative facilitation in language production. *Paper Presented at the 46th Annual Meeting of the Psychonomic Society*, Toronto, ON.

[B11] Butterworth B. (1989). “Lexical access in speech production,” in *Lexical Representations and Process*, ed. Marslen-Wilson W. (Cambridge, MA: MIT Press), 108–135.

[B12] Caramazza A. (1997). How many levels of processing are there in lexical access? *Cogn. Neuropsychol.* 14 177–208. 10.1080/026432997381664

[B13] Caramazza A. Laudanna A. Romani C. (1988). Lexical access and inflectional morphology. *Cognition* 28 297–332. 10.1016/0010-0277(88)90017-0 3359755

[B14] Clark H. H. (1973). The language-as-fixed-effect fallacy: a critique of language statistics in psychological research. *J. Verbal Learn. Verbal Behav.* 12 335–359. 10.1016/S0022-5371(73)80014-3

[B15] Costa A. Alario F.-X. Caramazza A. (2005). On the categorical nature of the semantic interference effect in the picture-word interference paradigm. *Psychon. Bull. Rev.* 12 125–131. 10.3758/BF03196357 15948287

[B16] Costa A. Caramazza A. Sebastian-Galles N. (2000). The cognate facilitation effect: implications for models of lexical access. *J. Exp. Psychol.* 26 1283–1296. 10.1037/0278-7393.26.5.1283 11009258

[B17] Damian M. F. Bowers J. S. (2003). Locus of semantic interference in picture-word interference tasks. *Psychon. Bull. Rev.* 10 111–117. 10.3758/BF03196474 12747497

[B18] Damian M. F. Martin R. C. (1999). Semantic and phonological codes interact in single word production. *J. Exp. Psychol.* 25 345–361. 10.1037/0278-7393.25.2.345 10093206

[B19] Dell G. S. (1986). A spreading-activation theory of retrieval in sentence production. *Psychol. Rev.* 93 283–321. 10.1037/0033-295X.93.3.283 3749399

[B20] Dell G. S. Reich P. A. (1981). Stages in sentence production: an analysis of speech error data. *J. Verbal Learn. Verbal Behav.* 20 611–629. 10.1016/S0022-5371(81)90202-4

[B21] Dobel C. Gumnior H. Bölte J. Zwitserlood P. (2007). Describing scenes hardly seen. *Acta Psychol.* 125 129–143. 10.1016/j.actpsy.2006.07.004 16934737

[B22] Dohmes P. Zwitserlood P. Bölte J. (2004). The impact of semantic transparency of morphologically complex words on picture naming. *Brain Lang.* 90 203–212. 10.1016/S0093-934X(03)00433-4 15172538

[B23] Eyelink II (2004). *[Apparatus, and Software].* Mississauga, ON: SR Research.

[B24] Fei-Fei L. VanRullen R. Koch C. Perona P. (2005). Why does natural scene categorization require little attention? Exploring attentional requirements for natural and synthetic stimuli. *Vis. Cogn.* 12 893–924. 10.1080/13506280444000571

[B25] Finkbeiner M. Caramazza A. (2006). Lexical selection is not a competitive process: a reply to La Heij et al. (2006). *Cortex* 42 1032–1035. 10.1016/S0010-9452(08)70210-7

[B26] Garrett M. F. (1980). “Levels of processing in sentence production,” in *Language Production Speech and Talk* Vol. 1 ed. Butterworth B. (London: Academic Press).

[B27] Glaser W. R. Düngelhoff F. (1984). The time course of picture-word interference. *J. Exp. Psychol. Hum. Percept. Perform.* 10 640–654. 10.1037/0096-1523.10.5.640 6238124

[B28] Glaser W. R. Glaser M. O. (1989). Context effects in Stroop-like word and picture processing. *J. Exp. Psychol.* 118 13–42. 10.1037/0096-3445.118.1.13 2522504

[B29] Goldrick M. (2006). Limited interaction in speech production: chronometric, speech error, and neuropsychological evidence. *Lang. Cogn. Process.* 21, 817–855. 10.1037/0096-3445.118.1.13

[B30] Gumnior H. Bölte J. Zwitserlood P. (2006). A chatterbox is a box: morphology in German word production. *Lang. Cogn. Process.* 21 920–944. 10.1080/016909600824278

[B31] Hemera Photo Objects (n.d.). *Volume I – III.[Computer Software].* Québec: Hemera Publisher.

[B32] Humphreys G. W. Lloyd-Jones T. J. Fias W. (1995). Semantic interference effects on naming using a postcue procedure: tapping the links between semantics and phonology with pictures and words. *J. Exp. Psychol.* 21 961–980. 10.1037/0278-7393.21.4.961

[B33] Humphreys G. W. Riddoch M. Quinlan P. T. (1988). Cascade processes in picture identification. *Cogn. Neuropsychol.* 5 67–104. 10.1080/02643298808252927

[B34] Janssen N. (2013). Response exclusion in word–word tasks: a comment on Roelofs, Piai and Schriefers. *Lang. Cogn. Process.* 28 672–678. 10.1080/01690965.2012.746715

[B35] Janssen N. Bi Y. Caramazza A. (2008). A tale of two frequencies: determining the speed of lexical access for Mandarin Chinese and English compounds. *Lang. Cogn. Process.* 23 1191–1223. 10.1080/01690960802250900

[B36] Jescheniak J. D. Hantsch A. Schriefers H. (2005). Context effects on lexical choice and lexical activation. *J. Exp. Psychol.* 31 905–920. 10.1037/0278-7393.31.5.905 16248741

[B37] Jescheniak J. D. Matushanskaya A. Mädebach A. Müller M. M. (2014). Interference from semantically related context pictures in single picture naming – Evidence for competitive lexical selection. *Psychon. Bull. Rev.* 21 1294–1300. 10.3758/s13423-014-0606-5 24687732

[B38] Jescheniak J. D. Oppermann F. Hantsch A. Wagner V. Mädebach A. Schriefers H. (2009). Do perceived context pictures automatically activate their phonological code? *Exp. Psychol.* 56 56–65. 10.1027/1618-3169.56.1.56 19261579

[B39] Jescheniak J. D. Schriefers H. (1998). Discrete serial versus cascaded processing in lexical access in speech production: further evidence from the coactivation of near-synonyms. *J. Exp. Psychol.* 24 1256–1274. 10.1037/0278-7393.24.5.1256

[B40] Koester D. Schiller N. O. (2008). Morphological priming in overt language production: electrophysiological evidence from Dutch. *Neuroimage* 42 1622–1630. 10.1016/j.neuroimage.2008.06.043 18674626

[B41] Koester D. Schiller N. O. (2011). The functional neuroanatomy of morphology in language production. *Neuroimage* 55 732–741. 10.1016/j.neuroimage.2010.11.044 21109010

[B42] La Heij W. Dirkx J. Kramer P. (1990). Categorical interference and associative priming in picture naming. *Br. J. Psychol.* 81 511–525. 10.1111/j.2044-8295.1990.tb02376.x

[B43] La Heij W. Heikoop K. W. Akerboom S. Bloem I. (2003). Picture naming in picture context: semantic interference or semantic facilitation? *Psychol. Sci.* 45 49–62.

[B44] Lakens D. (2013). Calculating and reporting effect sizes to facilitate cumulative science: a practical primer for t-tests and ANOVAs. *Front. Psychol.* 4:863 10.3389/fpsyg.2013.00863 PMC3840331 24324449

[B45] Levelt W. J. M. (1989). *Speaking: From Intention to Articulation.* Cambridge, MA: MIT Press.

[B46] Levelt W. M. Roelofs A. Meyer A. S. (1999). A theory of lexical access in speech production. *Behav. Brain Sci.* 22 1–38. 10.1017/S0140525X99001776 11301520

[B47] Levelt W. M. Schriefers H. Vorberg D. Meyer A. S. Pechmann T. Havinga J. (1991). The time course of lexical access in speech production: a study of picture naming. *Psychol. Rev.* 98 122–142. 10.1037/0033-295X.98.1.122

[B48] Lüttmann H. Zwitserlood P. Böhl A. Bölte J. (2011a). Evidence for morphological composition at the form level in speech production. *J. Cogn. Psychol.* 23 818–836. 10.1080/20445911.2011.575774

[B49] Lüttmann H. Zwitserlood P. Bölte J. (2011b). Sharing morphemes without sharing meaning: production and comprehension of German verbs in the context of morphological relatives. *Can. J. Exp. Psychol.* 65 173–191. 10.1037/a0023794 21728406

[B50] Mahon B. Z. Costa A. Peterson R. Vargas K. A. Caramazza A. (2007). Lexical selection is not by competition: a reinterpretation of semantic interference and facilitation effects in the picture-word interference paradigm. *J. Exp. Psychol.* 33 503–535. 10.1037/0278-7393.33.3.503 17470003

[B51] Mahon B. Z. Navarrete E. (2014). The Critical difference in models of speech production: a response to Roelofs and Piai. *Cortex* 52 123–127. 10.1016/j.cortex.2013.12.001 24525244

[B52] Martin N. Gagnon D. A. Schwartz M. F. Dell G. S. Saffran E. M. (1996). Phonological facilitation of semantic errors in normal and aphasic speakers. *Lang. Cogn. Process.* 11 257–282. 10.1080/016909696387187

[B53] Melinger A. Abdel Rahman R. (2004). Investigating the interplay between semantic and phonological distractor effects in picture naming. *Brain Lang.* 90 213–220. 10.1016/S0093-934X(03)00434-6 15172539

[B54] Meyer A. S. Damian M. F. (2007). Activation of distractor names in the picture-picture interference paradigm. *Mem. Cogn.* 35 494–503. 10.3758/BF03193289 17691148

[B55] Meyer A. S. Schriefers H. H. (1991). Phonological facilitation in picture-word interference experiments: effects of stimulus onset asynchrony and types of interfering stimuli. *J. Exp. Psychol.* 17 1146–1160. 10.1037/0278-7393.17.6.1146

[B56] Meyer A. S. Sleiderink A. M. Levelt W. M. (1998). Viewing and naming objects: eye movements during noun phrase production. *Cognition* 66 B25–B33. 10.1016/S0010-0277(98)00009-2 9677766

[B57] Meyer A. S. Wheeldon L. van der Meulen F. Konopka A. (2012). Effects of speech rate and practice on the allocation of visual attention in multiple object naming. *Front. Psychol.* 3:39 10.3389/fpsyg.2012.00039 PMC3282304 22363310

[B58] Morey R. D. (2008). Confidence intervals from normalized data: a correction to Cousineau (2005). *Tutor. Q. Methods Psychol.* 4 61–64.

[B59] Morsella E. Miozzo M. (2002). Evidence for a cascade model of lexical access in speech production. *J. Exp. Psychol.* 28 555–563. 10.1037/0278-7393.28.3.555 12018507

[B60] Navarrete E. Costa A. (2005). Phonological activation of ignored pictures: further evidence for a cascade model of lexical access. *J. Mem. Lang.* 53 359–377. 10.1016/j.jml.2005.05.001

[B61] Oppermann F. Jescheniak J. D. Görges F. (2014). Resolving competition when naming an object in a multiple-object display. *Psychon. Bull. Rev.* 21 78–84. 10.3758/s13423-013-0465-5 23761213

[B62] Oppermann F. Jescheniak J. D. Schriefers H. (2008). Conceptual coherence affects phonological activation of context objects during object naming. *J. Exp. Psychol.* 34 587–601. 10.1037/0278-7393.34.3.587 18444758

[B63] O’Séaghdha P. G. Frazer A. K. (2014). The exception does not rule: attention constrains form preparation in word production. *J. Exp. Psychol.* 40 797–810. 10.1037/a0035576 PMC4102258 24548328

[B64] Peterson R. R. Savoy P. (1998). Lexical selection and phonological encoding during language production: eidence for cascaded processing. *J. Exp. Psychol.* 24 539–557. 10.1037/0278-7393.24.3.539

[B65] Raaijmakers J. W. Schrijnemakers J. C. Gremmen F. (1999). How to deal with The language-as-fixed-effect fallacy: common misconceptions and alternative solutions. *J. Mem. Lang.* 41 416–426. 10.1006/jmla.1999.2650

[B66] Rapp B. Goldrick M. (2000). Discreteness and interactivity in spoken word production. *Psychol. Rev.* 107 460–499. 10.1037/0033-295X.107.3.460 10941277

[B67] Ratcliff R. (1979). Group reaction time distributions and an analysis of distribution statistics. *Psychol. Bull.* 86 446–461. 10.1037/0033-2909.86.3.446 451109

[B68] Rayner K. (2009). Eye movements and attention in reading, scene perception, and visual search. *Q. J. Exp. Psychol.* 62 1457–1506. 10.1080/17470210902816461 19449261

[B69] Roelofs A. (1992). A spreading-activation theory of lemma retrieval in speaking. *Cognition* 42 107–142. 10.1016/0010-0277(92)90041-F 1582154

[B70] Roelofs A. (1997). The WEAVER model of word-form encoding in speech production. *Cognition* 64 249–284. 10.1016/S0010-0277(97)00027-9 9426503

[B71] Roelofs A. (2003). Goal-referenced selection of verbal action: modeling attentional control in the Stroop task. *Psychol. Rev.* 110 88–125. 10.1037/0033-295X.110.1.88 12529058

[B72] Roelofs A. (2004). Comprehension-based versus production-internal feedback in planning spoken words: a Rejoinder to Rapp and Goldrick (2004). *Psychol. Rev.* 111 579–580. 10.1037/0033-295X.111.2.579

[B73] Roelofs A. (2008a). Tracing attention and the activation flow in spoken word planning using eye movements. *J. Exp. Psychol.* 34 353–368. 10.1037/0278-7393.34.2.353 18315411

[B74] Roelofs A. (2008b). Dynamics of the attentional control of word retrieval: analyses of response time distributions. *J. Exp. Psychol.* 137 303–323. 10.1037/0096-3445.137.2.303 18473661

[B75] Roelofs A. Baayen H. (2002). Morphology by itself in planning the production of spoken words. *Psychon. Bull. Rev.* 9 132–138. 10.3758/BF03196269 12026945

[B76] Roelofs A. Piai V. (2011). Attention demands of spoken word planning: a review. *Front. Psychol.* 2:307 10.3389/fpsyg.2011.00307 PMC3209602 22069393

[B77] Roelofs A. Piai V. Schriefers H. (2013). Selection by competition in word production: rejoinder to Janssen (2013). *Lang. Cogn. Process.* 28 679–683. 10.1080/01690965.2013.770890

[B78] Schriefers H. Meyer A. S. Levelt W. J. (1990). Exploring the time course of lexical access in language production: picture-word interference studies. *J. Mem. Lang.* 29 86–102. 10.1016/0749-596X(90)90011-N

[B79] Starreveld P. A. La Heij W. (1995). Semantic interference, orthographic facilitation, and their interaction in naming tasks. *J. Exp. Psychol.* 21 686–698. 10.1037/0278-7393.21.3.686

[B80] Starreveld P. A. La Heij W. (1996). Time-course analysis of semantic and orthographic context effects in picture naming. *J. Exp. Psychol.* 22 896–918. 10.1037/0278-7393.22.4.896

[B81] Stemberger P. (1985). “An interactive activation model of language production,” in *Progress in the Psychology of Language* Vol. 1 ed. Ellis A. W. (Hillsdale, NJ: Lawrence Erlbaum Associates Inc.), 143–186.

[B82] Zwitserlood P. (1994). “Access to phonological form representations in language comprehension and production,” in *Perspectives on Sentence Processing*, eds Clifton C. Frazier L. Rayner K. (Hillsdale, NY: Lawrence Erlbaum).

[B83] Zwitserlood P. Bölte J. Dohmes P. (2002). Where and how morphologically complex words interplay with naming pictures. *Brain Lang.* 81 358–367. 10.1006/brln.2001.2530 12081405

