# Supplementary material for: Investigating the flow of information during speaking: the impact of morpho-phonological, associative, and categorical picture distractors on picture naming
Source: Front Psychol. 2015 Oct 12;6:1540. doi: 10.3389/fpsyg.2015.01540 (PMC4600906; doi:10.3389/fpsyg.2015.01540)
Supplement: Supplementary file 1 [file Data_Sheet_1.DOCX]

# Appendix A Material Experiment 1

| Target | +A+M | +C+M | +A-M | +C-M | unrelated |
| --- | --- | --- | --- | --- | --- |
| Bierdeckel  (beer mat) | Bierkiste  (beer box) | Tankdeckel (fuel tank cap) | Tischdecke (tablecloth) | Kronkorken (crown cap) | Schlüsselanhänger (key fob) |
|  | Bierglas (beer glas) | Topfdeckel (lid) | Weinglas (wine glas) | Untersetzer (table mat) | Schildkröte (turtle) |
| Bierdose (beer can) | Bierkrug (beer mug) | Keksdose (biscuit box) | Fernsehzeitung (TV journal) | Weinflasche (wine bottle) | Zahnpasta (tooth paste) |
|  | Bierflasche (beer bottle) | Steckdose (socket) | Aschenbecher (ash tray) | Sektglas (champagne glass) | Schlauchboot (life raft) |
| Einkaufskorb (shopping basket) | Einkaufswagen (trolley) | Picknickkorb (picnic basket) | Strichcode (bar code) | Aktenkoffer (executive case) | Telefonhörer (phone receiver) |
|  | Einkaufstüte (shopping bag) | Maulkorb (muzzle) | Sparschwein (piggybank) | Handtasche (handbag) | Luftballon (balloon) |
| Fußball (football) | Fußspur (footprints) | Basketball (basketball) | Schiedsrichter (referee) | Bowlingkugel (bowling ball) | Erdbeere (strawberry) |
|  | Fußmatte (doormat) | Federball (shuttlecock) | Torwart (goalkeeper) | Hockeyschläger (shinny) | Goldfisch (goldfish) |
| Gartenstuhl (lawn chair) | Gartenschlauch (garden hose) | Schaukelstuhl (rocking chair) | Schwimmbecken (swimming pool) | Schuhregal (shoe rack) | Billiardkugel (billiard ball) |
|  | Gartenzwerg (garden gnome) | Bürostuhl (office chair) | Rasenmäher (lawn mower) | Schreibtisch (desk) | Zahnbürste (tooth brush) |
| Kaffeekanne (coffeepot) | Kaffeemühle (coffee mill) | Thermoskanne (thermos flask) | Teebeutel (tea bag) | Zuckerdose (sugar bowl) | Flugzeug (airplane) |
|  | Kaffeebohne (coffee bean) | Gießkanne (watering can) | Milchaufschäumer (milk frother) | Schöpfkelle (scoop) | Gasmaske (gas mask) |
| Kinderwagen (pushchair) | Kindersitz (child seat) | Lastwagen (lorry) | Babybrei (baby food) | Dreirad (tricycle) | Marienkäfer (ladybird) |
|  | Kinderstuhl (high chair) | Bollerwagen (trolley) | Strampelanzug (sleepers) | Schubkarre (wheel barrow) | Rollschuh (skates) |
| Kleiderschrank (cupboard) | Kleiderständer (hallstand) | Schuhschrank (shoe locker) | Bademantel (bathrobe) | Herrendiener (valet stand) | Schallplatte (record) |
|  | Kleiderbügel (hanger) | Kühlschrank (fridge) | Boxershorts (shorts) | Schublade (drawer) | Nagellack (nail polish) |
| Mülleimer (bin) | Müllbeutel (rubbish bag) | Putzeimer (cleaning bucket ) | Kehrblech (dustpan) | Benzinkanister (petrol can) | Bügelbrett (ironing board) |
|  | Mülltonne (bin) | Farbeimer (paint bucket) | Staubwedel (feather duster) | Papierkorb (wastebasket) | Telefonzelle (phone box) |
| Regenschirm (umbrella) | Regenjacke (raincoat) | Fallschirm (parachute) | Gummistiefel (wellingtons) | Spazierstock (walking stick) | Puppenhaus (doll's house) |
|  | Regenbogen (rainbow) | Gleitschirm (paraglider) | Wachsjacke (jacket) | Strohhut (straw hat) | Riesenrad (big wheel) |
| Sonnenbrille (sun glasses) | Sonnenmilch (sunmilk) | Taucherbrille (diving mask) | Strandmatte (beach mat) | Kontaktlinse (contact lens) | Lenkrad (steering wheel) |
|  | Sonnenfinsternis (eclipse of the sun) | Schwimmbrille (swimming mask) | Eiswaffel (ice-cream wafer) | Augenklappe (patch) | Handtuch (towel) |
| Taschenlampe (torch) | Taschenrechner (pocket calculator) | Stehlampe (standard lamp) | Isomatte (camping mat) | Glühbirne (electric bulb) | Kopfhörer (headphones) |
|  | Taschenuhr (pocket watch) | Öllampe (oil lamp) | Schlafsack (sleeping bag) | Kronleuchter (chandelier) | Malkasten (paint-box) |
| Tennisschläger (tennis racket) | Tennisball (tennis ball) | Golfschläger (golf club) | Ascheplatz (cinder court) | Einrad (unicycle) | Schaukelpferd (rocking horse) |
|  | Tennisplatz (tennis court) | Badminton­schläger (badminton racket) | Schiedsrichterstuhl (referee's chair) | Billardqueue (billiard cue) | Pfeffermühle (pepper mill) |
| Wäscheklammer (peg) | Wäscheständer (clotheshorse) | Büroklammer (paperclip) | Küchentuch (cloth) | Sicherheitsnadel (safety pin) | Leuchtturm (lighthouse) |
|  | Wäschespinne (rotary clothes drier) | Haarklammer (hairgrip) | Bügeleisen (flat iron) | Schraubzwinge (screw clamp) | Vogelscheuche (scarecrow) |
| Waschmaschine (washing machine) | Waschbecken (wash basin) | Kaffeemaschine (coffee maker) | Wäscheleine (clothesline) | Gasherd (gas stove) | Spielautomat (slot machine) |
|  | Waschmittel (detergent) | Nähmaschine (sewing-machine) | Weichspüler (fabric softener) | Staubsauger (vacuum cleaner) | Sprungbrett (diveboard) |

Roelofs (2008) formulated an exception, allowing for the incidental cascading to word-form information. One and the same concept may activate multiple word forms, as is the case for near synonyms (e.g., *sofa* and *couch*).

2 German compounds are written without spaces.

3 We use the term morpho-phonological overlap to signal that the target constituent overlaps phonologically with the distractor constituent. The phonological overlap constitutes at the same time a free morpheme. Morpho-phonological overlap is different from pure phonological overlap (Roelofs & Baayen, 2002; Zwitserlood, Bölte & Dohmes, 2000).

4 We do not report F2-analyses in this study because targets were repeated over conditions and not nested under conditions (Clark, 1973; Raaijmakers, Schrijnemaker & Gremmen, 1999). Linear mixed effects models that have been suggested as alternative to F1 and F2-analyses converged only without random slopes. The LME-analyses corroborated the reported results, but we do not report them here because we fell that the low number of trials per condition renders questionable the results of such analyses (Barr, Levy, Scheepers & Tily, 2013).

5 We used SPPS to compute $\eta_{p}^{2}$ and a spreadsheet provided by Lakens (2013) to compute $\eta_{g}^{2}$.

6 One participant was excluded due to the number of outliers.
